# Eagles enter rotor‐swept zones of wind turbines at rates that vary per turbine

**DOI:** 10.1002/ece3.7911

**Published:** 2021-07-21

**Authors:** Christopher J. W. McClure, Brian W. Rolek, Melissa A. Braham, Tricia A. Miller, Adam E. Duerr, Jennifer D. McCabe, Leah Dunn, Todd E. Katzner

**Affiliations:** ^1^ The Peregrine Fund Boise Idaho USA; ^2^ Conservation Science Global West Cape May New Jersey USA; ^3^ Division of Forestry and Natural Resources West Virginia University Morgantown West Virginia USA; ^4^ US Geological Survey, Forest and Rangeland Ecosystem Science Center Boise Idaho USA

**Keywords:** Bald Eagle, collision risk, Golden Eagle, mitigation, wind energy, wind power, wind turbine

## Abstract

There is increasing pressure on wind energy facilities to manage or mitigate for wildlife collisions. However, little information exists regarding spatial and temporal variation in collision rates, meaning that mitigation is most often a blanket prescription. To address this knowledge gap, we evaluated variation among turbines and months in an aspect of collision risk—probability of entry by an eagle into a rotor‐swept zone (hereafter, “probability of entry”). We examined 10,222 eagle flight paths identified and recorded by an automated bird monitoring system at a wind energy facility in Wyoming, USA. Probabilities of entry per turbine–month combination were 4.03 times greater in some months than others, ranging 0.15 to 0.62. The overall probability of entry for the riskiest turbine (i.e., the one with the greatest probability of entry) was 2.39 times greater than the least‐risky turbine. Our methodology describes large variation across turbines and months in the probability of entry. If subsequently combined with information on other sources of variation (i.e., weather, topography), this approach can identify risky versus safe situations for eagles under which cost of management, curtailment prescriptions, and collision risk can be simultaneously minimized.

## Graphical abstract

Rates that eagles entered rotor‐swept zones of wind turbines varied by turbine across a wind farm in Wyoming, USA. Such variation demonstrates that curtailment criteria should vary per turbine and season.

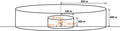

## 1 INTRODUCTION

Wind energy technology has advanced considerably in past decades (Gibson et al., 2017; Veers et al., 2019), yet ecological challenges such as wildlife fatalities hinder wind power from reaching its full potential (Katzner et al., 2019). There is spatial and temporal variation in these ecological challenges. For example, some individual wind turbines are especially dangerous (i.e., they are identical in form to other nearby turbines, but the location at which they are installed makes them dangerous to volant wildlife; de Lucas & Perrow, 2017; Marques et al., 2014). Such locations might be avoided by wind power developers in efforts to lessen potential collision fatalities. Further, wind conditions are highly variable, creating temporal variation in the risk of collision at a given turbine (Barrios & Rodríguez, 2004). Researchers have consequently recommended that turbines installed in dangerous locations be rendered inactive during parts of the year, or under weather conditions when fatalities are most likely (Barrios & Rodríguez, 2004; Gartman et al., 2016; Smallwood et al., 2009; Smallwood & Karas, 2009).

An alternative to shut down during certain predetermined periods is informed curtailment (sensu Allison et al., 2017), also known as “shutdown on demand” or “selective stopping,” in which individual wind turbines are curtailed when animals are present in the immediate area and estimated to be at high risk of collision (Allison et al., 2017; BirdLife International, 2015). However, informed curtailment is difficult and expensive to implement because of the technological difficulty of detecting birds at risk, and knowledge gaps regarding where and when mortality most frequently occurs. Despite this, one of the few tests of informed curtailment involved human observers at 13 wind power facilities in southern Spain, which achieved a 50% reduction in mortality of Griffon Vultures (*Gyps fulvus*; de Lucas et al., 2012).

Automated technology for bird monitoring has potential to optimize curtailment decisions to reduce collisions and maximize power production of turbines. Radar has been used in several instances to monitor birds (Aschwanden et al., 2018; Jenkins et al., 2018; Plonczkier & Simms, 2012; Tomé et al., 2017), as has camera‐based technology (Aschwanden et al., 2015; Collier et al., 2012; May et al., 2012), and a hybrid camera‐radar system (Niemi & Tanttu, 2020). At Top of the World Wind Power Facility (hereafter, “Top of the World”) in Wyoming, USA, automated curtailment using a camera‐based system (IdentiFlight^®^) reduced eagle fatalities relative to a nearby control facility, but markedly increased the number of curtailments (McClure et al., 2021).

When implementing informed curtailment, risk assessment generally is uniform across an entire wind power facility and management and mitigation only rarely take into account turbine or site‐specific properties that likely influence probability of collision. This is problematic because the monetary costs of a poorly designed approach to curtailment can be significant enough to make it economically unviable. Here, we used data collected from IdentiFlight units over 11 months at Top of the World to examine potential spatial and temporal variation in collision risk of birds at a wind facility known to be dangerous to large soaring raptors. To examine an important component of collision risk, we calculated the probability an eagle that approached within 150 m of a given turbine subsequently entered the area swept by that turbine's blades (i.e., “the rotor‐swept zone”). We call this component of risk “probability of entry.” The probability of entry is only one facet of overall collision risk, which also involves the likelihood a bird enters a wind farm, then approaches a turbine, and once within a rotor‐swept zone, fails to evade the turbine blades (May, 2015). We predicted variation in probability of entry across space and time such that it varies by interacting temporal (by month) and turbine‐specific processes. We quantified this variation and used it to inform the value of curtailment criteria that vary among turbines and over time, to provide insight into the relevance of a uniform approach to curtailment.

## 2 METHODS

### 2.1 Study site

**FIGURE 1:**
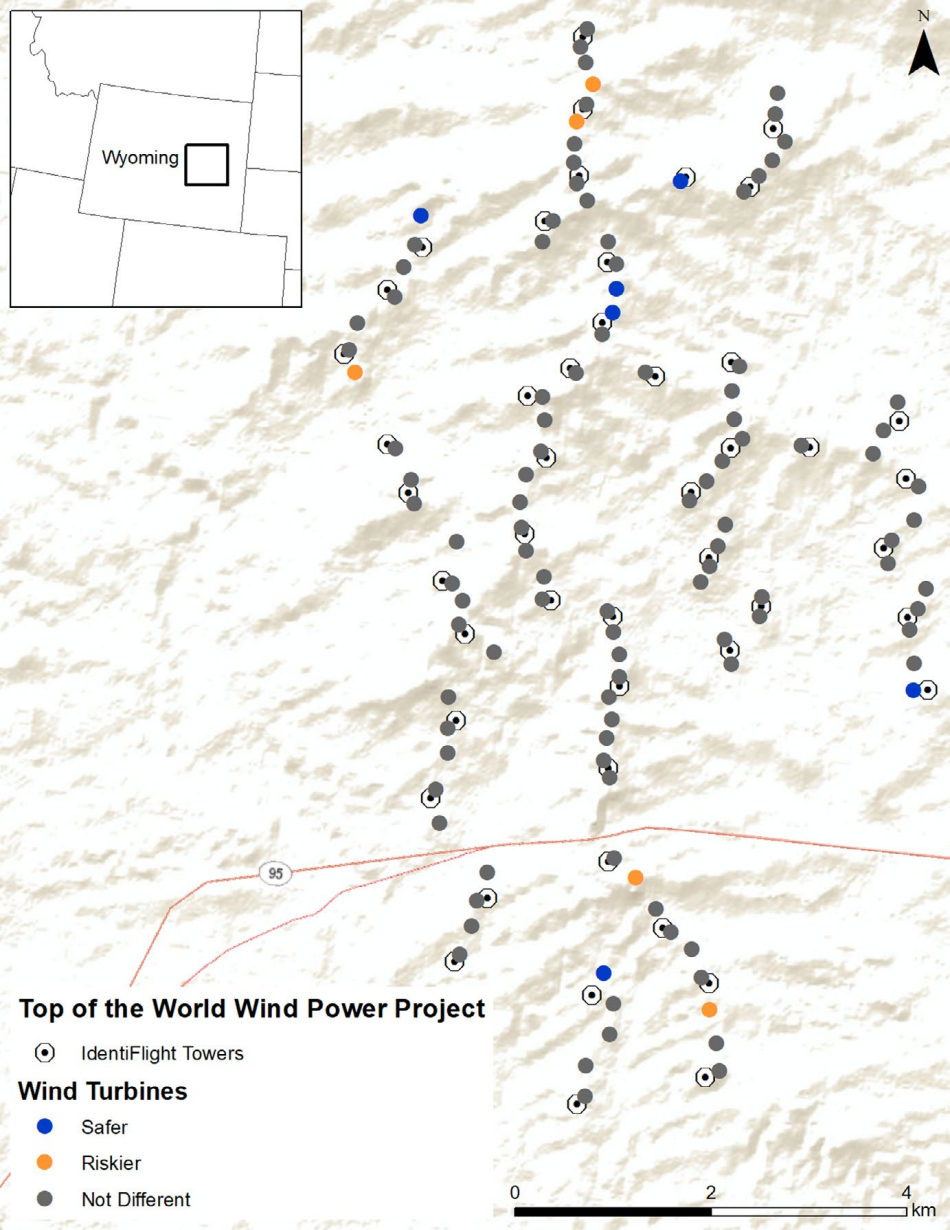
Top of the World Wind Power Project in Wyoming. Colors represent whether a turbine is statistically safer (blue)—lower probability of rotor‐swept zone entry—or riskier (orange)—higher probability of rotor‐swept zone entry—than the average turbine (gray)

Operated by Duke Energy Renewables, Top of the World contains 44 Siemens 2.3‐MW, 101‐m rotor diameter wind turbines and 66 General Electric 1.5‐MW, 77‐m rotor diameter turbines (Figure 1). Both styles of turbines have a hub height of 80 m above ground, making maximum blade height 131.5 m and 119 m, respectively. For this study, we defined rotor‐swept zones as three‐dimensional cylinders centered at the hub height of a given turbine with the radii equal to the buffer distance provided by IdentiFlight. The height of the cylinder was determined by the known maximum and calculated minimum height of the wind turbine blades. We obtained the known maximum height of turbine blades from the public online Federal Aviation Administration obstructions database (https://oeaaa.faa.gov/). We calculated the minimum height of the wind turbine by subtracting twice the blade length from the maximum height of the turbine. Golden Eagles (*Aquila chrysaetos*) are common at Top of the World, with Bald Eagles (*Haliaeetus leucocephalus*) also occurring. Duke Energy began human‐based informed curtailment in 2014 and transitioned to IdentiFlight during 2018–2019.

### 2.2 IdentiFlight

IdentiFlight is a camera system designed to detect and classify birds in flight. It is integrated with an artificial intelligence system to determine, in real time, whether any turbines within the camera system's viewshed should be curtailed. Each camera system (hereafter: “IdentiFlight unit”) consists of a ring of eight stationary Wide Field of View (WFoV) cameras and one High Resolution Stereo Camera (HRSC) mounted on a Pan and Tilt Unit. The WFoV cameras detect moving objects and track them. The HRSC subsequently estimates the line‐of‐sight distance to the object and takes photographs every 100 ms (10/s). IdentiFlight units at Top of the World use an algorithm to classify, as eagle or noneagle, objects in those photographs that are <1,000 m from the unit. While tracking, IdentiFlight units record the three‐dimensional flight paths of each bird, with data recorded as positions (latitude, longitude, and altitude) collected at approximately one‐second intervals. See McClure et al. (2018) for further details.

At Top of the World, IdentiFlight units were placed on their own towers seven to 10 m tall and interspersed throughout the facility in a configuration that provided visual coverage for all turbines. Over the course of the study, 47 IdentiFlight units were deployed (Figure 1). The first units were installed in May 2018 with more installed in July 2018, January 2019, and March 2019. Upon installation, these units began tracking birds, recording flight paths, and deciding whether curtailment was warranted, although they were not actually controlling curtailments until 1 August 2018.

### 2.3 Curtailment criteria

**FIGURE 2:**
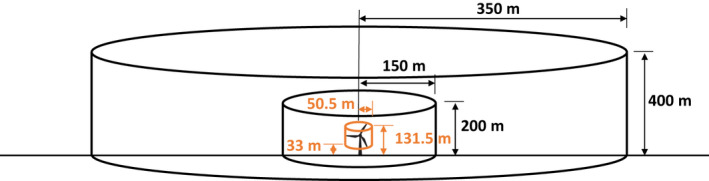
Diagram of the curtailment criteria applied at Top of the World Wind Power Facility. The curtailment criteria use two virtual cylinders (black) to determine when curtailment is triggered. The inner‐most cylinder (orange) depicts the rotor‐swept zone of a Siemens 2.3‐MW, 101‐m rotor diameter wind turbine. Curtailment never occurs unless an eagle is within the outer cylinder. Between the inner and outer black cylinders, curtailment is triggered using a time to collision threshold, which was 10 s during August 2018 to March 2019. Curtailments were always triggered if eagles were within the inner black cylinder. Birds must have been classified as eagles (≥90% confidence in classification of eagle vs. noneagle) to trigger curtailment decisions

The current IdentiFlight curtailment regime uses, as threshold criteria, two virtual cylinders centered on each turbine hub (Figure 2). Curtailment of a given turbine is never considered unless there is an eagle (≥90% confidence in automated classification of eagle vs. noneagle) within the outer cylinder. When a bird is detected within the outer cylinder but outside of the inner cylinder, curtailment is ordered if the flight trajectory indicates an eagle will enter the rotor‐swept zone within a specified amount of time (i.e., time to collision; Figure 2). Curtailment is always ordered if an eagle is detected within the inner cylinder (Figure 2). The radii and heights of the cylinders and the time to collision threshold can be tailored based on the risk tolerance of facility managers, but these values apply equally across the entire facility and are not tailored per turbine or season. Initially, the radii of the inner and outer cylinders were set at 200 and 400 m, respectively, and the time to collision set to 30 s. Criteria were made less restrictive—allowing eagles to approach closer before curtailment—in August 2018 by reducing the radii of the inner and outer cylinders to 150 m and 350 m, respectively, and the time to collision to 10 s. The heights of the inner and outer cylinders were 200 and 400 m, respectively, and did not change during the entire study.

### 2.4 Data

The curtailment criteria thus serve as a filter to remove observed flights that were never near enough to a turbine for there to be risk of collision. We obtained data from the date of first installation, May 2018, until 31 March 2019 from each individual IdentiFlight unit. Because IdentiFlight units were installed in phases and because eagles do not approach all turbines with equal frequency, we collected different amounts of data from different units. These data consisted of the individual one‐second positions for each bird flight that triggered a curtailment order. We only examined flights that approached within 150 m of a turbine. The change in curtailment criteria in August 2018 therefore should not influence our study.

### 2.5 Analysis

#### 2.5.1 Turbines and months

We built a mixed‐effects logistic regression model to examine temporal and turbine‐specific patterns in risk of eagles entering rotor‐swept zones of turbines. The input data we considered were from flight paths of eagles (as classified by IdentiFlight) that approached within 150 m of at least one turbine. Our model used a binary response variable describing whether a flight entered (1) or did not enter (0) the rotor‐swept zone of the nearest turbine. We included a random effect for turbine–month combination in a two‐way interaction‐effects ANOVA formulation (Kéry, 2010). There were no fixed effects in the model. See Appendix S1 for model code. The model thus estimated, for each turbine in each month, the probability that an eagle that flew within 150 m of a turbine would enter the rotor‐swept zone. For this analysis, we used each approach of a turbine within 150 m as a data point. Therefore, when a flight passed within 150 m of multiple turbines, we treated each of those turbines as separate opportunities for entry into a rotor‐swept zone. Because 91.5% of flight tracks passed within 150 m of a single turbine, and 8% approached two turbines, there was little risk of pseudoreplication within tracks, and thus, we did not attempt to control for multiple approaches by individual tracks. In instances where two IdentiFlight units tracked the same eagle at the same time, we used the data from the unit nearest to the eagle.

This modeling procedure allowed us to determine, for each approach of an eagle within 150 m, the month‐specific probability of that bird entering a rotor‐swept zone of a given turbine and then to statistically compare that to a reference probability level. Because the curtailment regime at Top of the World assumes that the probability of rotor‐swept zone entry is the same across space and time, we used the average probability of rotor‐swept zone entry across the entire study (number of rotor‐swept zone entries at all turbines/number of approaches within 150 m of any turbine) as the reference probability. We calculated the average probabilities per turbine as the mean of all turbines across all months and the average probabilities per month as the mean across turbines within that month. We calculated the difference between posterior draws of the average probability and those of each turbine, month, and combination thereof. We did this by subtracting the posterior draws from the average. Then, we calculated the probability that each turbine, month, and turbine–month combination was different from average using the proportion of differences between posterior draws that were greater than zero, that is, the probability of direction (Makowski et al., 2019). We considered a turbine or month to be statistically safer or riskier than average if the probability of direction was larger than 0.95 or less than 0.05, respectively.

We fitted the model with Bayesian methods implemented using JAGS (Plummer, 2003). We used the package jagsUI (Kellner, 2016) as an interface between JAGS and R (R Core Team, 2019), and we simulated three chains of 5,000 iterations, including a burn‐in of 500 and thinning rate of 2. We calculated the Gelman–Rubin statistic (Gelman & Rubin, 1992) and assessed the degree to which convergence of chains was achieved (i.e., parameters had an $\hat{R}$ < 1.1). We also viewed trace plots of posterior parameter chains to assess convergence. We used vague priors (uniform for means and variances) for all parameters (Kéry & Royle, 2016).

## 3 RESULTS

### 3.1 Probability of entry into the rotor‐swept zone

Of the 23,625 occurrences of eagle flight paths that met curtailment criteria, IdentiFlight recorded 10,222 within 150 m of a turbine. Of these, 3,015 entered rotor‐swept zones. The average probability of an eagle flying within 150 m of a wind turbine and subsequently entering its rotor‐swept zone (i.e., probability of entry) was therefore 29.5% (95% CRI = 28.6%–30.4%) across all turbines and months.

### 3.2 Ranges of probabilities of entry into the rotor‐swept zone

**FIGURE 3:**
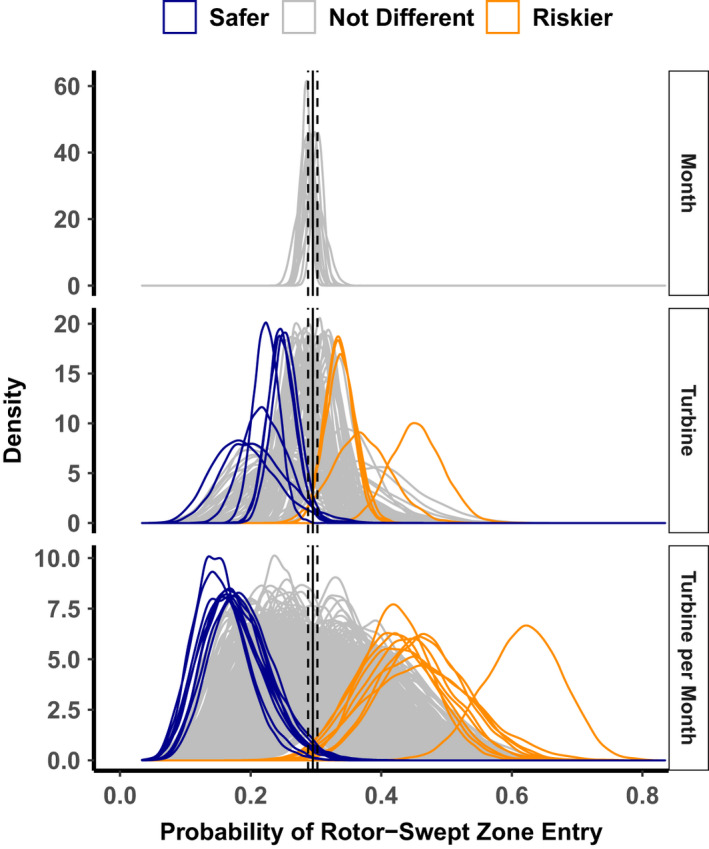
Density plots of draws from posterior distributions of probabilities of bird flights recorded by IdentiFlight entering the rotor‐swept zones of the nearest turbine. These estimates were calculated per turbine, month, and per turbine–month. Solid vertical lines indicate the average across the entire study, and dotted lines indicate the upper and lower limits of the 95% credible interval of that average. Colors indicate whether there is at least a 90% probability of the posterior distributions being different from the mean. Orange curves represent turbines, months, or combinations thereof having a statistically higher than average probability of rotor‐swept zone entry and are thus interpreted to be riskier than average. Conversely, blue curves represent turbines, months, or combinations thereof having a statistically lower than average probability of rotor‐swept zone entry and are considered safer than average

Across all turbines, the average probability of entry did not vary among months (Figure 3, Appendix S2). However, probability of entry varied among turbines, with five of 110 turbines being riskier than average (i.e., associated with a high probability of entry) and six being safer (i.e., associated with a low probability of entry; Figures 1 and 3; Appendix S3) based on our 0.05 or 0.95 probability of direction thresholds. The average probabilities of entry per turbine ranged from 19.0% (95% CRIs = 10.6%–29.3%; probability of direction = 0.976) to 45.4% (95% CRIs = 37.8%–53.4%; probability of direction = 0.00; Figures 1 and 3; Appendix S3). The modeled overall probability of entry for the riskiest turbine was 2.39 times greater than that of the least‐risky turbine.

**FIGURE 4:**
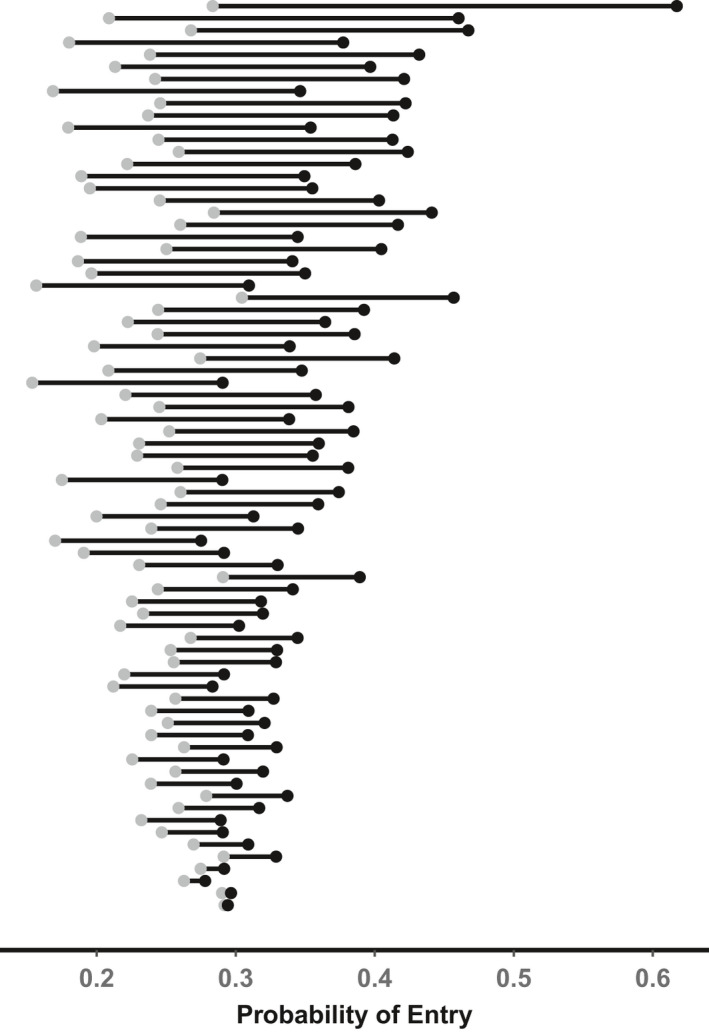
Ranges of probabilities of entry per month for each turbine that was monitored >1 month (*n* = 75). Each line represents one turbine, gray points depict the minimum monthly value, and black points represent the maximum monthly value

The average probabilities of entry per turbine–month combination ranged from 15.3% (95% CRIs = 8.3%–24.1%; probability of direction = 0.998) to 61.7% (95% CRIs = 50.0%–72.8%; probability of direction = 0.00; Figure 3; Appendix S4)—a 4.03 times difference. The standard deviation in the probability of entry among turbine months was 0.09 (95% CRIs = 0.08–0.10). There were 15 combinations of month and turbine that were safer than average, and 10 combinations of month and turbine that were riskier than average (Figure 3; Appendix S4). Individual turbines that were monitored >1 month ranged in monthly probability of entry by as much as 0.334 and as little as 0.002 (Figure 4; Appendix S4).

## 4 DISCUSSION

We quantify variation in an aspect of collision risk, the probability of entry, at a wind energy facility. Our work supports past studies that used fatalities to identify variation in risk among turbines (de Lucas, Ferrer, Bechard, et al., 2012; de Lucas et al., 2008; Smallwood et al., 2007). We expected seasonality in probability of entry across the facility, yet we found little difference in the average among months. However, substantial changes in monthly probability of entry per turbine indicate that although average probability of entry across our study site is stable throughout the year, it varies by turbine per month. Although some level of variation among turbines is to be expected, accurately quantifying and describing this variation has important potential to be used to improve risk management and curtailment efforts.

Site characteristics of turbines, wind conditions, and behavior of eagles all likely drive the heterogeneity in probability of entry we observed. Topography can influence wind patterns to create especially attractive, yet perilous wind conditions for birds (de Lucas & Perrow, 2017). Indeed, wind speed and direction further affect collision risk (Ferrer et al., 2012; de Lucas, Ferrer, & Janss, 2012). Vegetation, livestock management, and rodent control efforts can influence availability of prey across space and time (Allison et al., 2017; Gartman et al., 2016), thus changing the attractiveness of an area. Eagles engaging in certain behaviors, such as hunting and migrating, may also alter collision risk (de Lucas & Perrow, 2017; Marques et al., 2014). Both migratory and breeding Golden Eagles occur at Top of the World and shifts in turbine‐specific probability of entry might be due to seasonal changes in behavior. For example, Golden Eagles fly at lower altitudes when moving locally than during migration, putting local birds at a greater risk of collision (Katzner et al., 2012). Further, eagles may avoid turbines when actively migrating by adjusting flight altitude (Johnston et al., 2014).

Seasonal variation in weather is almost certainly a driver of some of the temporal variation we detected in probability of entry. In fact, weather patterns change seasonally, and those seasonal changes in weather can influence avian flight. For example, California Condors spend more time at flight altitudes in the rotor‐swept zone during cooler, winter months when flight is subsidized primarily by orographic updrafts, and less time at those altitudes in warmer, summer months when flight is subsidized primarily by thermal updraft (Poessel et al., 2018). Similarly, there is an important seasonal component to the flight altitudes of Golden Eagles in California (Duerr et al., 2019), which, again, is almost certainly driven by weather. Although we do not evaluate effects of weather on flight behavior directly, our analyses suggest a strong role for weather on collision risk of these eagles. As such, next steps for this analysis involve identifying the meteorological drivers of variation in collision risk for eagles at Top of the World and other wind energy facilities.

Some of the heterogeneity, or lack thereof, we observed was possibly due to the staggered implementation of automated curtailment over the course of the study. Not all turbines were covered by IdentiFlight units over the entire course of the study, and therefore, more extreme values of probability of entry might be observed if turbines were monitored over the course of an entire year. Further, our dataset spans a relatively short time frame and we do not know how stable these patterns are among years, for example, if certain turbines are always most dangerous during the same month every year. Longer‐term data would be needed to assess the annual reliability of these seasonal patterns.

Another important caveat is that probability of entry is only one facet of the process of an eagle colliding with a turbine blade (May, 2015). However, the probability of entry is especially relevant to modifying the curtailment decision because it addresses whether curtailment was warranted. Curtailment would ideally occur only when an eagle will definitely enter a rotor‐swept zone. In practice, one cannot know this for certain, so operators of wind power facilities make decisions to curtail when there is sufficiently high probability of entry. Our results demonstrate that probability of entry can vary across a wind facility, information that could be used to optimize curtailment criteria.

Variation in probability of entry among turbines provides inference into potential situations that might allow for tailoring curtailment criteria at any facility with turbine‐specific or temporal variation in collision risk. We noted variation in probability of entry among months and turbines, although because we only considered one year of data, we can draw limited inference from among‐month variation. Likewise, although some level of variation is to be expected, the range of probabilities of entry was substantial, varying by >two times per turbine (Figure 3). To meet the objective of minimizing energy loss and reducing risk to wildlife, curtailment criteria for turbines with higher than expected probabilities of entry could be more stringent, with shutdown initiated at greater distances or longer estimated time to collision. In contrast, some turbines were safer than average. In those cases, curtailment criteria might be eased, thus maintaining a constant probability of collision while allowing birds to approach closer before curtailment is triggered (Ferrer et al., 2012; Lucas, Ferrer, & Janss, 2012).

Existing curtailment strategies do not account for variation in risk among turbines. Examining variation in the probability of entry is an important step in establishing a protocol for modifying curtailment criteria across space and time. Further steps might be to identify drivers of probability of entry, as noted above, or to take a retrospective approach, determining which curtailment criteria would have optimized the trade‐off between power generation and collision risk. The spatial variation that we demonstrate can therefore be the foundation of techniques to minimize both collision risk to birds and the costs of operational mitigation of wind turbines.

## CONFLICT OF INTEREST

The authors declare no conflicts of interest.

## AUTHOR CONTRIBUTIONS

**Christopher J. W. McClure:** Conceptualization (equal); Formal analysis (lead); Funding acquisition (lead); Methodology (equal); Project administration (lead); Visualization (lead); Writing‐original draft (lead); Writing‐review & editing (supporting). **Brian W. Rolek:** Conceptualization (equal); Data curation (equal); Formal analysis (supporting); Writing‐review & editing (equal). **Melissa A. Braham:** Data curation (lead); Formal analysis (equal); Methodology (lead); Writing‐review & editing (equal). **Tricia A. Miller:** Conceptualization (equal); Writing‐review & editing (equal). **Adam E. Duerr:** Conceptualization (equal); Writing‐review & editing (equal). **Jennifer D. McCabe:** Conceptualization (equal); Writing‐review & editing (equal). **Leah Dunn:** Conceptualization (equal); Data curation (equal); Writing‐review & editing (equal). **Todd E. Katzner:** Conceptualization (equal); Supervision (equal); Writing‐review & editing (equal).

## Supporting information

Appendix S1‐S4

## Data Availability

Data available on dryad: https://doi.org/10.5061/dryad.r4xgxd2cs.
